# Machine learning approaches to supporting the identification of photoreceptor-enriched genes based on expression data

**DOI:** 10.1186/1471-2105-7-116

**Published:** 2006-03-08

**Authors:** Haiying Wang, Huiru Zheng, David Simpson, Francisco Azuaje

**Affiliations:** 1 School of Computing and Mathematics, University of Ulster, UK; 2 Department of Ophthalmology, Queen's University of Belfast, UK

## Abstract

**Background:**

Retinal photoreceptors are highly specialised cells, which detect light and are central to mammalian vision. Many retinal diseases occur as a result of inherited dysfunction of the rod and cone photoreceptor cells. Development and maintenance of photoreceptors requires appropriate regulation of the many genes specifically or highly expressed in these cells. Over the last decades, different experimental approaches have been developed to identify photoreceptor enriched genes. Recent progress in RNA analysis technology has generated large amounts of gene expression data relevant to retinal development. This paper assesses a machine learning methodology for supporting the identification of photoreceptor enriched genes based on expression data.

**Results:**

Based on the analysis of publicly-available gene expression data from the developing mouse retina generated by serial analysis of gene expression (SAGE), this paper presents a predictive methodology comprising several *in silico *models for detecting key complex features and relationships encoded in the data, which may be useful to distinguish genes in terms of their functional roles. In order to understand temporal patterns of photoreceptor gene expression during retinal development, a two-way cluster analysis was firstly performed. By clustering SAGE libraries, a hierarchical tree reflecting relationships between developmental stages was obtained. By clustering SAGE tags, a more comprehensive expression profile for photoreceptor cells was revealed. To demonstrate the usefulness of machine learning-based models in predicting functional associations from the SAGE data, three supervised classification models were compared. The results indicated that a relatively simple instance-based model (*KStar *model) performed significantly better than relatively more complex algorithms, e.g. neural networks. To deal with the problem of functional class imbalance occurring in the dataset, two data re-sampling techniques were studied. A random over-sampling method supported the implementation of the most powerful prediction models. The KStar model was also able to achieve higher predictive sensitivities and specificities using random over-sampling techniques.

**Conclusion:**

The approaches assessed in this paper represent an efficient and relatively inexpensive *in silico *methodology for supporting large-scale analysis of photoreceptor gene expression by SAGE. They may be applied as complementary methodologies to support functional predictions before implementing more comprehensive, experimental prediction and validation methods. They may also be combined with other large-scale, data-driven methods to facilitate the inference of transcriptional regulatory networks in the developing retina. Furthermore, the methodology assessed may be applied to other data domains.

## Background

Retinal photoreceptor cells, the specialized cells involved in light detection and phototransduction, are essential for mammalian vision. Many retinal diseases occur as a result of inherited dysfunction of the rod and cone photoreceptor cells. Photoreceptor degeneration, for example, constitutes an important cause of visual impairment affecting all age groups and ethnic backgrounds [1]. Development and maintenance of photoreceptor function in the retina requires appropriate regulation of gene expression, especially for genes specifically or highly expressed in photoreceptor cells during retinal development (photoreceptor-enriched genes). Comprehensive identification of photoreceptor-enriched gene expression patterns may have important implications in neurobiology, leading to a better understanding of molecular mechanisms of retinal development, the improvement of diagnosis of complex retinal diseases, and the identification of potential therapeutic targets [2].

Over the last decades, different experimental approaches have been developed to identify retinal disease genes. Using microarray data analysis, for example, Yoshida *et al. *[3] revealed that 43 genes, which are differentially expressed in the absence of *Nrl *(neural retina leucine zipper protein), are either associated with or are candidates for retinal diseases involving rod or cone photoreceptor dysfunction. Katsanis *et al. *[4] positioned 925 *expressed sequence tags *(ESTs) likely to be specifically or preferentially expressed in the retina. They also identified positional candidate genes for 42 of 51 uncloned retinopathies. The quality of the results was assessed by *reverse transcriptase-polymerase chain reaction *(RT-PCR). Recently, Blackshaw *et al. *[2] presented a comprehensive genomic analysis of mouse retinal development using *serial analysis of gene expression *(SAGE), followed by *in situ *hybridization (ISH) validation. Libraries were obtained from microdissected mouse photoreceptors from the retinal outer nuclear layers (ONL), retina from various mouse developmental stages and retina from the paired-homeodomain transcription factor Crx knockout mouse (Crx^-/-^) and its wild type counterpart (Crx^+/+^) at postnatal day (P)10, and from NIH3T3 mouse fibroblasts.

The SAGE-based expression analysis performed by Blackshaw *et al. *[2] has advantages over other RNA analysis methods. This technique uses a unique sequence tag of 13 or more bases isolated from a defined position within each transcript [5]. The basic concept of SAGE rests on two principles: (1) a short nucleotide sequence tag contains sufficient information to uniquely identify a transcript and (2) concatenated short sequence tags can be cloned to facilitate efficient sequencing analysis. Unlike RNA blotting and RT-PCR, SAGE is not limited to examining only a few known genes at a time. Unlike DNA microarray technology, the SAGE approach allows the simultaneous analysis of a large number of transcripts without *prior*, complete knowledge of the sequence of the genes [6].

In the study by Blackshaw *et al. *[7], the candidate photoreceptor-specific genes were selected by comparative analysis between SAGE libraries on the basis of four chosen criteria (see Results Section). This method, however, has shown relatively low true positive and true negative prediction rates. For example, out of 196 newly-identified photoreceptor-enriched (PR-enriched) tags, only approximately 20% meet all the four classification criteria and about 44% meet more than three of the four criteria. To further identify true PR-enriched tags, Blackshaw *et al*. experimentally validated the candidate tags through exhaustive ISH analysis. In this paper, we explore the feasibility of using computational approaches to support large-scale analysis of photoreceptor gene expression data. The main purpose of this study is to implement several *in silico *models to detect complex features and relationships encoded in the SAGE data, which may be used to predict functional associations. By way of illustration, this paper focuses on the classification of two functional classes of genes, which were studied and experimentally validated by Blackshaw *et al. *[7]: PR-enriched and non-PR-enriched genes. The main question was: Can machine learning-based classifiers be built to accurately distinguish PR-enriched from non PR-enriched genes solely based on patterns in the SAGE data? The potential benefits of this approach are two-fold. In situations for which there is insufficient biological knowledge, machine learning-based classifiers could be used to predict functional classes of genes. Secondly, if classifiers are demonstrated to effectively predict specific gene types, these empirically derived relationships could be used to derive biological significance.

In order to study temporal patterns of photoreceptor gene expression during retinal development, a two-way cluster analysis: clustering of tags and clustering of libraries were performed. While SAGE libraries were clustered using traditional hierarchical clustering method, SAGE tags were analysed by a newly developed Poisson model-based *k*-means algorithm (PoissonC) specifically designed for SAGE data [8]. To address one of the limitations of PoissonC, a *Figure of Merit *(FOM)-based approach [9] to estimating the number of clusters was proposed. The FOM is computed by first removing one experiment (library in our case), clustering genes based on the remaining data, and then measuring how the left-out library fits the expression patterns obtained from the other libraries. Such adaptations represent one of the aspects distinguishing our investigation from traditional clustering-based analyses. To explore the feasibility of machine-learning approaches to predicting functional associations encoded in the SAGE data generated from different developmental stages in mouse retina, three supervised classification methods were tested to predict the two functional classes investigated by Blackshaw *et al. *[7]: PR-enriched and non-PR-enriched genes. To deal with the problem of functional class imbalance occurring in the dataset, two data re-sampling techniques were studied. To adequately evaluate the performance of the supervised classifiers in imbalanced dataset, three predictive quality indicators that are independent of the class prior probabilities were implemented. To further assess the statistical significance of the computational approaches, a 100-run permutation test was implemented. The reader is referred to the section of Methods for a more detailed description of the data sets and techniques studied. The following section summarises relevant results.

## Results

### Clustering of SAGE libraries

**Figure 1:**
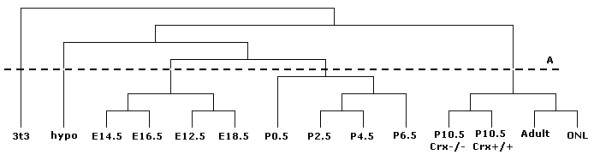
**A hierarchical tree for 14 SAGE libraries based on analysis of 1118 tags**. The hierarchical tree was generated based on 1118 tags highly expressed in the ONL library using Pearson correlation-based hierarchical clustering method. Five clusters are obtained when cutting the dendrogram at level A.

**Figure 2:**
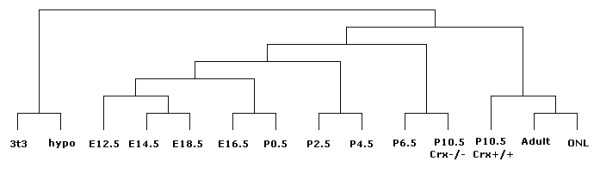
**A hierarchical tree for 14 SAGE libraries based on analysis of 261 PR-enriched tags**. This hierarchical tree provides further insights. The split at birth is less marked. The P10.5 Crx-/- library is clustered with the P6.5 library rather than with its wild type counterpart.

Figure 1 depicts a hierarchical tree for 14 SAGE libraries based on all 1118 tags highly expressed in the ONL library generated by agglomerative method with Pearson correlation as a similarity measure. In general, it reflects the relationship between the libraries based on their gene expression levels. As expected, libraries within the same developmental period are more closely related than libraries representing gene expression from other time points. Four embryonic libraries (E12.5, E14.5, E16.5, and E18.5) and four postnatal libraries (P0.5, P2.5, P4.5, and P6.5) are all grouped together. When we cut the dendrogram at the level A, five clusters are obtained. Two libraries belonging to non-retinal tissues (3t3 and hypo) are clearly separated from other clusters, which confirm that the SAGE libraries reflect tissue specificity. The embryonic libraries are split from the postnatal libraries, most likely reflecting the effect of the physiological changes occurring at birth on gene expression. Moreover, the 8 postnatal libraries are clustered into two groups, 4 of which are grouped together after P6.5. This might suggest that a critical time point occurs between P6.5 and P10.5 for the development of photoreceptor cells. In order to focus on photoreceptor cells, 14 SAGE libraries were clustered using the 261 PR-enriched tags, as shown in Figure 2. This provides further insights, for example the split at birth is less marked, which is consistent with evidence showing that terminal differentiation and functional activation of photoreceptor cells occurs at later stages [7]. The P10.5 Crx-/- library now clusters with the P6.5 library rather than with its wild type counterpart, as would be predicted from the essential function of Crx in photoreceptor cell type specification. This suggests that its loss has a greater effect on PR-enriched genes and their profile more closely resembles that of the immature P6.5 wild type retina.

### Clustering of SAGE tags

**Figure 3:**
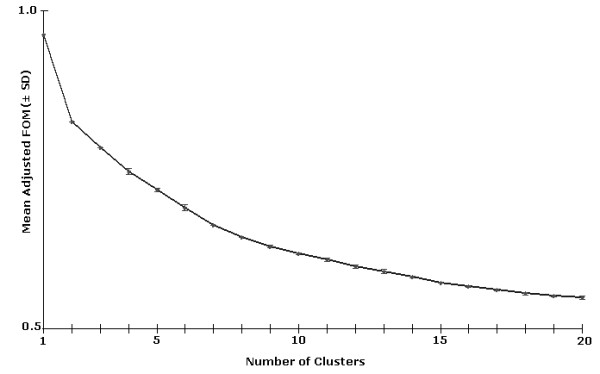
**The mean values of 5-runs adjusted FOM calculations against the number of clusters**. An open-source implementation of FOM provided by the Institute for Genomic Research (TIGR) [22] was used to calculate the adjusted FOM for *k*-means algorithm with Euclidean distance on normalised SAGE data. For a given tag, the abundance in each SAGE library was rescaled to make the sum of tag counts across all 14 libraries equal to one.

**Figure 4:**
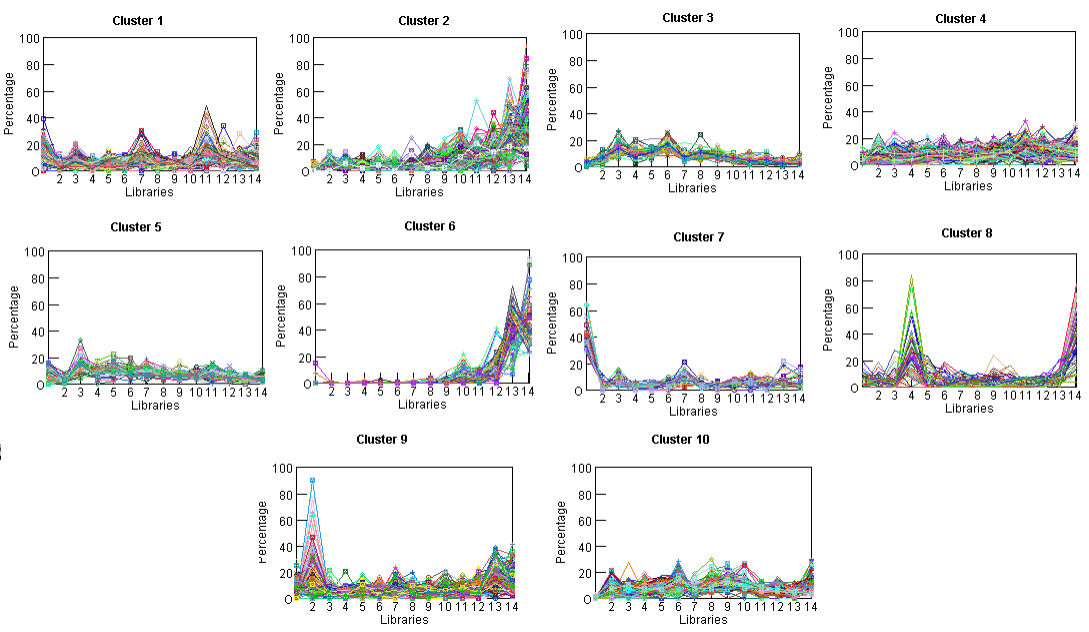
**Poisson model-based clustering analysis for photoreceptor gene expression using 10 clusters**. SAGE libraries are plotted on the x-axis. Numbers one to fourteen represent the fourteen SAGE libraries mentioned in Method Section, i.e. 1: hypo; 2: 3t3; 3: E12.5; 4: 14.5; 5: E16.5; 6: E18.5; 7: P0.5; 8: P2.5; 9: P4.5; 10: P6.5; 11: P10.5Crx-/-; 12: P10.5Crx+/+; 13: Adult; 14: ONL. Tag abundance is shown on the y-axis. Data were normalized before plotting. Each tag from the 14 libraries was rescaled to make the sum of the expression values equal to one. Different colors represent different tags.

Figure 3 shows 5-runs adjusted FOM values for the *k*-means algorithm with Euclidean distance on normalised SAGE data from 1 to 20 clusters. The value of adjusted FOM steeply decreases until the number of clusters is equal to 10. Afterwards the rate of decline with respect to the number of clusters is reduced. The results for a 10-cluster analysis using the PoissonC algorithm are shown graphically in Figure 4. Table 1 lists the number of tags within each cluster, the number of tags previously known to be PR-enriched/non-PR enriched, as well as the number of tags identified as PR-enriched/non-PR enriched by Blackshaw *et al*. [7]. The description of the cluster profiles is also given in Table 1. The clustering results from a Euclidean-based clustering model are given in the supplementary materials.

In general, the temporal expression patterns observed in this SAGE data reflect previously characterized photoreceptor gene expression patterns in the developing retina. For example, genes associated with tags in Clusters 2 and 6, whose expression consistently increases throughout postnatal development and reaches their highest value in the adult retina, are highly likely to be expressed in developing photoreceptors. Sixty out of 63 tags previously known to be PR-enriched and 134 out of 197 tags identified to be PR-enriched by Blackshaw *et al. *[7] fell within these two clusters, highlighting that this is an important feature which can be used to identify PR-enriched genes. A closer examination of these two clusters revealed that many tags were mapped to genes with known functions relevant to phototransduction and visual formation. For example, 8 tags associated with *rhodopsin *gene and 2 tags linked to *rod photoreceptor *were grouped together in Cluster 6. Four tags mapped to *guanine nucleotide binding proteins *(G-protein) [10], which are involved as modulators or transducers in various transmembrane signalling systems, were all found in Cluster 2. Apart from those tags directly related to the visual process, some tags associated with genes with other functions such as *peripherin-2 *and *rod outer segment membrane protein 1 *(ROM1) were also found. It has been shown that these genes are involved in maintaining the integrity of photoreceptor outer segment and are therefore critical for rod photoreceptor viability and regulation of disk morphogenesis [11].

Interestingly, 8 out of the 10 known non-PR-enriched tags were found in Cluster 9, having peak expression values occurring within 3T3 fibroblast cells. This might confirm that those genes that have higher expression levels in non-retina tissues are unlikely to become PR-enriched genes. Cluster 7 exhibits similar expression profiles of genes strongly expressed in adult hypothalamus. Although the characteristics of all 27 tags in this cluster were not investigated by Blackshaw *et al. *[7], our analysis indicates that these genes are unlikely to be selectively expressed in photoreceptors based on the observations derived from Cluster 9.

Clusters 3 and 5, whose peak expression values occurred during embryonic development, also offered relevant insights. Genes that fell within these two clusters generally have higher expression levels in the mouse retina before P6.5 with expression gradually decreasing throughout later postnatal development. Examples includes macrophage migration inhibitory factor gene in Cluster 5, which plays an important role in T-cell activation and may contribute to regulation of retinal inflammation and its local immunity [12]. Its expression starts at E12.5, peaks at E16.5, and significantly decreases in the adult. In clusters 3 and 5, a total of 12 tags were examined by Blackshaw *et al. *[7], ten out of 12 resulted non-PR enriched. The expression profile exhibited by these two clusters may serve as template for the detection of non-PR enriched genes.

Unlike the other gene clusters, the expression levels of genes within Clusters 1, 4, 8 and 10 did not follow a consistent trend throughout retinal development. Forty-six tags associated with various PR-enriched genes were found in these four different clusters, reflecting the heterogeneity of photoreceptor gene expression patterns on the basis of the onset and peak time of expression [2]. Unlike most of the photoreceptor genes (e.g. Rhodopsin) whose expression values are very low at birth and dramatically increase throughout postnatal retinal development, some of the PR-enriched genes exhibit totally different expression patterns. In Cluster 10, for example, expression of *NeuroD *is high at early embryonic stages, peaking at P4.5 and decreases significantly in the adult. This might reflect a dual function of *NeuroD *in cell specification and in regulation of rod photoreceptor survival [13]. In Cluster 8, *Mertk*, a c-mer proto-oncogene tyrosine kinase, exhibits an embryonic expression profile with peak at E14.5 and is selectively expressed in mature photoreceptor cells. Previous studies have shown that mutations in *Mertk *are responsible for retinal dystrophy [14]. The diversity of photoreceptor expression profiles may reflect the fact that some of PR-enriched genes are involved in more than one biological process.

### Supervised functional classification of tags

The goal was to determine whether a tag represents a PR-enriched or a non-PR-enriched gene given a set of SAGE libraries associated with each tag. Among the 1118 tags representing at least 0.01% of the total expression in the ONL library, 261 tags have been identified as PR-enriched genes, which exhibit diverse and complex expression patterns. This highlights the difficulties in using *in silico *methods to detect key relationships encoded in the SAGE data. Such complexities are further stressed when the original data are projected on 3-dimensional space using well-known mapping methods, i.e. *Principal Component Analysis *and *Sammon's mapping*, which clearly indicate that the two classes are not linearly separable from each other [see Additional file 1].

Based on a comprehensive analysis of the expression of tags previously known to be PR-enriched, Blackshaw *et al. *[7] introduced four criteria for the selection of candidate PR-enriched genes:

1. Tissue specific (criterion 1): the number of tags in either the hypothalamus or 3T3 libraries is less than 2.

2. Developmentally regulated (criterion 2): the sum of tags in the P10.5 crx+/+ (wild type), adult and ONL libraries divided by the sum of tags in the E12.5, E14.5, and E16.5 libraries is greater than 10-fold.

3. Crx dependent (criterion 3): tags are present at a level greater than 1.6-fold higher in the P10.5 crx+/+ library compared to the library of crx-/- mice.

4. ONL enriched (criterion 4): tags are present at an equal or greater number in the ONL library compared to the whole adult retina library.

We encoded the SAGE dataset using these criteria to study significant associations between the two functional classes and these criteria. The *Apriori algorithm *was applied to extract association rules from the SAGE data. This algorithm, which was proposed by Agrawal and Skrikant [15], is a well-known association rule learning algorithm. Given a dataset the *Apriori algorithm *is able to generate association rules that have support and confidence levels greater than user-specified values. A list of all association rules induced from the 324 tags, together with their support and confidence levels, are given in Table 2. The distribution of these 324 tags on the basis of their compliance with the four criteria is given in the additional file [see Additional file 1].

The results obtained showed that a high rate of true positives was observed for genes corresponding to tags meeting more than two of the four criteria. However, when applying these criteria to perform supervised classification, poor prediction results were obtained. For example, only 57 out of 261 PR-enriched tags met all four criteria. From the 196 tags identified by Blackshaw *et al. *[7] as PR-enriched, only about 20% met all four criteria and around 44% met more than three criteria. When applying the four criteria to the data individually, a relatively low rate of true negatives was obtained. For instance, 22 out of 63 non-PR-enriched genes met criterion 1, 11 met criterion 2, 28 met criterion 3, and 40 met criterion 4. Therefore, these classification criteria do not represent accurate and robust rules for the classification.

**Figure 5:**
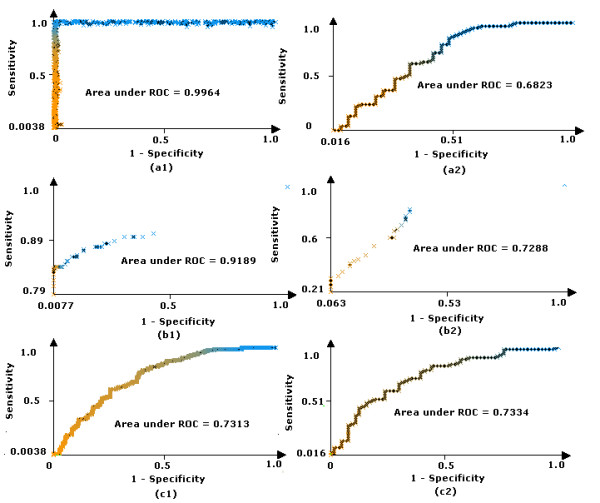
**The ROC curves for three classifiers using random over-sampling and under-sampling methods**. Figure (a1) KStar with over-sampling method; (a2) KStar with under-sampling method; (b1) C4.5 with over-sampling method; (b2) C4.5 with under-sampling method; (c1) MLP with over-sampling method; (c2) MLP with under-sampling method. The blue and yellow lines in ROC curves represent the different threshold values which are used to generate ROC.

Tables 3 and 4 show the prediction results from 10-fold cross validation for three supervised classifiers using random over- and under-sampling methods respectively. The section of Methods provides a description of these models. For each classifier, the overall classification accuracy (*Ac*), along with the precision (*Pr*), *Se *and *Sp *for each class, were calculated. Precision is defined as the proportion of predictions that are correct. The mathematical definitions of these metrics are given in the additional file [see Additional file 1]. The corresponding ROC graphs are depicted in Figure 5. For each ROC graph, the area under ROC curve (*AUC*) was calculated. The *AUC *has been suggested as a reliable and robust measure for classification performance [16]. A higher value of *AUC *is associated with a classifier that is both effective and robust, i.e. it presents a better average classification performance across different prediction (decision) thresholds.

Tables 3 and 4 indicate that the classifiers built on data derived from over-sampling methods provided better results than those derived from under-sampling methods in terms of *Ac*, *Se *and *Sp*. The advantages of random over-sampling techniques can be further demonstrated by the *AUC *values shown in Figure 5. This may be explained by the fact that a random under-sampling strategy may throw away potentially useful data. In addition, we found that the relatively simple *KStar *[17] algorithm can outperform more complex models such as MLP.

To investigate the effect of class distribution on the classifier, we varied the class distribution using the data over-sampling technique. Table 5 shows prediction results for the KStar classifier with different class distributions. A 10-fold cross validation procedure was carried out to estimate the true classification error. The corresponding ROC curves are given in the additional file [see Additional file 1]. The KStar with balanced class sample distribution achieved the best results.

To further assess the statistical significance of our computational approaches and their predictive performance, a 100-run permutation test was implemented. For each permutated dataset, the results were significantly worse than the one generated using the original data in terms of *Ac*, *Se*, and *Sp*, strongly indicating that the relationship between the data and the labels may be reliably learned by the proposed classifiers. For example, when implementing the permutation test for the *KStar *model on a balanced dataset and 10-fold cross validation, the random classifiers never performed better than the (original) prediction model built. The obtained average values of *Ac*, *Se *and *Sp *(for class PR-enriched) were significantly lower than the results shown in Table 3 (*p *< 0.01). Similar results were obtained when we performed a permutation test for other classifiers on the dataset with different class distributions. The complete results of our permutation test can be found in the additional file [see Additional file 2].

## Discussion

As the most accessible part of the central nervous system (CNS) and as a highly ordered laminar structure, the retina offers unique opportunities to study both the development and physiology of the CNS. This paper described several *in silico *approaches, including unsupervised and supervised models, to supporting large-scale analysis of photoreceptor gene expression by SAGE.

By clustering SAGE libraries, a hierarchical tree reflecting the relationship between the libraries was obtained. Libraries from adjacent developmental periods were generally grouped together as expected. However, significant discontinuities were identified at the time of birth and between P6.5 and P10.5, highlighting important developmental periods. By clustering SAGE tags, a more comprehensive expression profile for photoreceptor cells was revealed. It confirmed that most of the PR-enriched genes may be successfully clustered. These genes have lower expression levels before birth with expression dramatically increasing throughout postnatal development. Nevertheless, a closer examination of the clustering results revealed that photoreceptor expression patterns are highly heterogeneous and not separable by linear methods. The diversity of photoreceptor expression profiles reflects the variability in onset of expression which can occur early in development or when photoreceptors undergo terminal differentiation.

With regard to the clustering of SAGE tags, different algorithms with different distance metrics have been previously proposed. For example, it has been suggested that by modelling SAGE data with Poisson statistics better results can be achieved. However, it is relatively computationally expensive and Poisson-based distance has only been assessed as part of the *k*-means algorithm, which exhibits several limitations that hinder its performance. In this study, we proposed a framework to estimate the number of clusters for PoissonC based on the calculation of an adjusted FOM value. Nevertheless, there is a need to further expand clustering-based studies for SAGE data. For instance, an important question is how to incorporate Poisson-based distance into other clustering methods such as hierarchical clustering and self-organizing maps.

To demonstrate the usefulness of machine learning-based models in predicting functional associations from the SAGE data, a comprehensive comparative assessment of three supervised classification models was presented. The results indicated that a relatively simple instance-based model (*KStar *model) performed significantly better than relatively more complex algorithms, e.g. neural networks. This may be partly explained by the fact that neural network-based prediction models typically require larger amounts of high quality training data. Given the limited amount of SAGE data available, neural network-based algorithms may not be recommended. Nevertheless, the application of different types of machine learning approaches, including state-of-the-art classifiers such as *Support Vector Machine*, deserves further investigations.

Due to the imbalanced class distribution of the SAGE data, two re-sampling techniques: random over-sampling and under-sampling methods were studied. The results indicated that over-sampling strategies may provide more accurate predictions than under-sampling methods. This result seems to contradict some studies previously published in the literature [18]. However, other studies have suggested that when there is a significant disproportion in the number of samples belonging to each class, random under-sampling methods could actually ignore many potentially relevant data. Investigations of more sophisticated re-sampling techniques [16] will be part of our further research. We also intend to further address some of the limitations exhibited by such techniques, such as the predictive bias imposed by the incorporation of partially disjoint data sets during cross-validation.

The results suggest that, machine learning approaches such as *KStar *model may be useful for many purposes. For example, it can be applied as an inexpensive, user-friendly technique to support functional predictions in the retina before applying more comprehensive validation methods. It can be used to effectively select candidate genes for studies of retinal development and function.

## Conclusion

The methodology assessed represents an efficient and relatively inexpensive approach for supporting functional predictions. The techniques discussed in this paper can be in principle regarded as a generic framework, scalable to other types of data and biological functions. They can support functional predictions *prior *to the application of more comprehensive, integrative validation methods. They can be used to effectively select candidate genes for further studies and may also be combined with other large-scale, data-driven methods to facilitate the inference of transcriptional regulatory networks in the developing retina.

## Methods

### The dataset under study

The database under study was generated by the Cepko group at Harvard Medical School [7]. This database comprises a total of 14 murine SAGE libraries from different tissues and developmental stages, including mouse NIH-3T3 fibroblast cells, adult hypothalamus, developing retina at 2 day intervals from embryonic day (E) 12.5 to postnatal day (P) 6.5, P10.5 retinas from the paired-homeodomain gene crx knockout mouse (crx-/-) and from wild type (crx+/+) littermates, adult retina and microdissected outer nuclear layer (ONL). A total of 50000 – 60000 tags were sequenced from each tissue library, resulting in a dataset large enough to encompass all genes expressed at moderate or high levels in photoreceptor cells.

In order to control for sampling variability and to allow expression examination via ISH, we focused on 1118 tags whose abundance levels represent at least 0.01% of the total mRNA expression in the ONL library as done by Blackshaw *et al*. [7]. The distribution of these tags within the two retinal functional classes is given in Table 6. Under the Class column, TRUE and FALSE stand for genes validated by Blackshaw *et al*. using ISH; KNOWN and KNOWN FALSE represent genes validated prior to Blackshaw *et al*. 's study; N.D. stands for tags not validated in Blackshaw *et al*.'s study; and UNKNOWN includes tags which did not correspond to any identifiable transcript [7].

### Clustering methods

A central problem in the design of clustering models is the selection of a distance function to measure differences between expression profiles. Traditional approaches include the Euclidean distance and Pearson correlation coefficient. It has been shown that with regard to classification of SAGE libraries, Pearson correlation-based clustering analysis may detect significantly similar groups of genes [19]. On the other hand, for clustering of SAGE tags, different algorithms with different distance metrics have been proposed. Buckhaults *et al. *[20] adopted hierarchical cluster analysis with centered correlation similarity metric to support the identification of diagnostic SAGE tags. Becquet *et al. *[21] used self-organising tree algorithm to perform clustering analysis on human SAGE data. Based on the implementation of several distance metrics into the *k*-means procedure, Cai *et al. *[8] argued that Poisson-based distances are more appropriate and reliable for analysing SAGE data than traditional approaches. Thus, in the present study, SAGE libraries were clustered by hierarchical clustering with Pearson correlation as a measure of similarity, while SAGE tags were clustered using a *k*-means clustering algorithm based on the Poisson distance function (PoissonC) specifically designed for SAGE data [8]. The reader is referred to [8] for a detailed description of this algorithm.

Like other *k*-means models, one limitation of the PoissonC algorithm is that it requires users to specify a *priori *the number of clusters to be detected in the data. To deal with this problem, we used the FOM to estimate the optimal number of clusters encoded in the data [9]. The lower the FOM value is, the higher the predictive power of the algorithm. To compensate for a possible statistical bias when using many clusters, an adjusted FOM was implemented. A detailed description of the calculation of the adjusted FOM can be found in [9].

Due to the computational cost and characteristics of the PoissonC algorithm, the calculation of the adjusted FOM based on PoissonC may be a time-consuming process. It has been suggested that the patterns encoded in the SAGE data revealed by the clusters under different algorithms roughly agree with each other. Moreover, the performance (i.e. classification effectiveness) of clustering algorithms can be improved when the algorithms are applied to normalized data [8]. Thus, we proposed the following framework to estimate the appropriate cluster numbers for PoissonC:

1. Calculate the adjust FOM value using traditional *k*-means algorithm, in which the Euclidean distance was used to measure similarity on normalised SAGE data. For a given tag, the abundance in each SAGE library was rescaled to make the sum of tag counts across all 14 libraries equal to one. In our application the adjusted FOM was calculated for a range of numbers of clusters, from 1 to 20.

2. Draw the FOM value against the number of clusters graph.

3. Estimate the optimal number of clusters based on the graph

4. Use the number obtained in last step as an input to perform PoissonC-based clustering analysis.

We adopted an open-source implementation of FOM provided by the Institute for Genomic Research (TIGR) [22]

### Supervised classification methods

Three different classification models were implemented using the freely available Weka package [23]: *KStar*, C4.5 decision tree, and *multilayer perceptron *(MLP) neural network model. KStar is an instance-based classifier [17]. Based on information theory, it uses an entropy-based distance function to compute the similarity between two cases. The use of entropy as a distance measure provides a robust approach to handling different types of attributes such as symbolic and real-valued data [17]. We tested MLP models with different architectures without observing prediction performances significantly different to the results reported in this paper. The representative MLP results included here were obtained from a model with one hidden layer consisting of 8 neurones. The learning epochs for MLP was set to 500. For C4.5 algorithm, the minimum number of instances per leaf was equal to 2. A more detailed description of learning parameters for these models can be found in additional file [see Additional file 1].

These three models were assessed as classifiers for PR-enriched genes on the basis of SAGE data. To estimate the true classification error rate, a 10-fold cross validation was applied. To further assess the statistical validity of our computational approaches, 100-run permutation tests were performed in this study, i.e.: the labels for each tag were randomly shuffled, classifiers were then implemented, their prediction quality was assessed and this process was repeated for a number of permuted datasets. By counting the times the permuted datasets produced better results than the classifier built on the original dataset, the statistical significance was then established.

A key challenge was to address the class imbalance exhibited by the dataset available – as demonstrated by the highly skewed distribution of tags (Table 6). The predictive performance of traditional machine learning models may be significantly compromised when dealing with this type of data [24]. The problem is how to effectively distinguish patterns belonging to the *minority *class, i.e. non-PR-enriched class, from the *majority *class under consideration. To deal with this problem, two data re-sampling techniques were studied: *random under-sampling *and *random over-sampling*. The former method randomly eliminates majority class tags to achieve a balanced dataset. The latter randomly replicates minority class samples until a balanced class distribution is reached [25].

Another crucial problem is how to evaluate the performance of classifiers in imbalanced dataset. Traditional techniques include the calculation of classification accuracy based on a confusion matrix. However, it is known that when classes are imbalanced, these two metrics may offer misleading conclusions because they are strongly biased to favour the majority class [16]. For example, if we suppose that all non-PR-enriched tags were incorrectly classified as PR-enriched, a classifier would still be able to achieve a high classification accuracy (around 80%). Such a classifier, however, would be irrelevant. Thus, what is needed is a classification quality indicator that is independent of the class *prior *probabilities. It has been suggested that the true negative rate (also known as *specificity*, *Sp*), true positive rate (also known as *sensitivity*, *Se*), and *Receiver Operating Characteristic *(ROC) graphs are three appropriate metrics to assess the quality of a classifier in the presence of class imbalances [16]. It is evident that a true-positive prediction in the PR-enriched category is a true-negative in the non-PR-enriched category. Therefore, the values of *Se *and *Sp *for non-PR-enriched were omitted in Tables 3 to 5.

## Authors' contributions

HW and HZ co-designed the study, implemented the models, and drafted the manuscript. DS provided advice on biological aspects and co-wrote the paper. FA co-designed the study and co-wrote the manuscript. All authors read and approved the final manuscript.

## Supplementary Material

Additional File 1 **the supplementary material** Click here for file

Additional File 2 **The complete results for the permutation test** Click here for file

## Figures and Tables

**Table 1:** Summary of Poisson-based analysis for the SAGE data.

Cluster	No. of Tags	Description of cluster profile	No. of Tags validated *prior *to Blackshaw *et al. *[7]		No. of Tags validated by Blackshaw *et al. *[7]	
			
			PR-enriched	non-PR-enriched	PR-enriched	non-PR-enriched
1	105	Varying expression	0	0	5	1
2	196	Increasing during postnatal development	26	1	114	13
3	53	Peak expression occurred during embryonic development	0	1	1	6
4	280	Flat	1	0	19	11
5	63	Peak expression occurred during embryonic development	0	0	0	4
6	102	Increasingly sharp during late postnatal development	34	0	20	4
7	27	Peak expression in adult hypothalamus	0	0	0	0
8	55	Peak expression occurred around P4.5 and ONL	1	0	4	2
9	132	Peak expression in NIH-3T3 fibroblast cells	1	8	16	5
10	132	Varying expression	1	0	18	7

**Table 2:** A list of rules extracted from 324 tags using the *Apriori algorithm*.

Enriched in PR? <= criterion 4 (234:72.2%, 0.829)
Enriched in PR? <= criterion 1 (218:67.3%, 0.899)
Enriched in PR? <= criterion 3 (177:54.6%, 0.842)
Enriched in PR? <= criterion 2 (175:54.0%, 0.937)
Enriched in PR? <= criterion 4 & criterion 1 (165:50.9%, 0.909)
Enriched in PR? <= criterion 4 & criterion 3 (129:39.8%, 0.884)
Enriched in PR? <= criterion 4 & criterion 2 (117:36.1%, 0.949)
Enriched in PR? <= criterion 1 & criterion 3 (126:38.9%, 0.929)
Enriched in PR? <= criterion 1 & criterion 2 (143:44.1%, 0.944)
Enriched in PR? <= criterion 3 & criterion 2 (105:32.4%, 0.943)
Enriched in PR? <= criterion 4 & criterion 1 & criterion 3 (92:28.4%, 0.946)
Enriched in PR? <= criterion 4 & criterion 1 & criterion 2 (100:30.9%, 0.96)
Enriched in PR? <= criterion 4 & criterion 3 & criterion 2 (70:21.6%, 0.**971**)
Enriched in PR? <= criterion 1 & criterion 3 & criterion 2 (90:27.8%, 0.944)
Enriched in PR?<= criterion 4 & criterion 1 & criterion 3 & criterion 2 (59:18.2%, 0.966)

Each rule is shown in the following format: *Enriched in PR? <= criterion 1 & criterion 2 & ... &criterion n *where the rule is interpreted as "for tags that meet *criterion 1 *through *criterion n*, they are likely to be PR-enriched genes." The numbers shown at the end of each rule indicate the number of tags to which the rule applies (**Support**) and the proportion of those tags for which the rule is true (**Confidence**). **Support **is reported both as number of tags and percentage of total tags, separated by a colon.

**Table 3:** Prediction results of 10-fold cross validation for three classifiers using random over-sampling method. The total number of SAGE tags analyzed is 522, in which 261 are PR-enriched. Each tag is represented by 14 SAGE libraries.

Method	*Ac *(%)	PR-enriched			non-PR-enriched
		
		*Pr *(%)	*Se *(%)	*Sp *(%)	*Pr *(%)
KStar	91.2	99.5	82.8	99.6	85.2
C4.5	91.0	97.3	84.3	97.7	86.1
MLP	66.5	61.6	87.4	45.6	78.3

**Table 4:** Prediction results of 10-fold cross validation for three classifiers using random under-sampling method. The total number of SAGE tags analyzed is 126, in which 63 are PR enriched. Each tag is represented by 14 SAGE libraries.

Method	*Ac *(%)	PR-enriched			non-PR-enriched
		
		*Pr *(%)	*Se *(%)	*Sp *(%)	*Pr*(%)
Kstar	61.9	61.5	63.5	60.3	62.3
C4.5	66.7	66.7	66.7	66.7	66.7
MLP	65.1	60.9	84.1	46.0	74.4

**Table 5:** The effect of class distribution on the performance of the classifier.

Class distribution (PR-enriched : non-PR-enriched)	*Ac *(%)	PR enriched			non-PR enriched
		
		*Pr *(%)	*Se *(%)	*Sp *(%)	*Pr *(%)
1:1	91.2	99.5	82.8	99.6	85.2
2:1	89.8	95.7	85.8	94.9	83.6
3:1	89.1	94.3	88.9	89.6	80.7
4:1	84.1	88.8	91.2	58.3	64.6
Original (261:63)	81.2	86.2	91.2	39.7	52.1

The class distribution is obtained based on random over sampling method. A 10-fold cross validation was carried out to estimate the true classification error. Class distribution is represented as the number of PR-enriched tags against the number of non-PR-enriched tags. There are 261 PR-enriched and 63 non-PR-enriched tags in the original dataset. In all resampled datasets, the number of PR-enriched tags is as also equal to 261.

**Table 6:** Distribution of tags under study in terms of functional classification

Class		Number of tags	
PR-enriched	TRUE	197	261
	KNOWN	64	
non-PR-enriched	FALSE	53	63
	KNOWN FALSE	10	
N.D.		708	
UNKNOWN		86	
Total		1118	

Under the Class column, TRUE and FALSE stand for genes validated by Blackshaw *et al*. using ISH; KNOWN and KNOWN FALSE represent genes validated prior to Blackshaw *et al*. 's study; N.D. stands for tags not validated in Blackshaw *et al*.'s study; and UNKNOWN includes tags which did not correspond to any identifiable transcript [7].
