# Supplementary material for: Machine learning approaches to supporting the identification of photoreceptor-enriched genes based on expression data
Source: BMC Bioinformatics. 2006 Mar 8;7:116. doi: 10.1186/1471-2105-7-116 (PMC1421439; doi:10.1186/1471-2105-7-116)
Supplement: Additional File 1 — the supplementary material [file 1471-2105-7-116-S1.pdf]

Supplementary Section

Machine learning approaches to supporting the identification of photoreceptor-enriched genes based on expression data

Haiying Wang[a], Huiru Zheng[a], David Simpson[b], Francisco Azuaje[a]
[a]School of Computing and Mathematics, University of Ulster, UK, [b]Department of Ophthalmology, Queen's University of Belfast, UK

S.1 PoissonC algorithm

Based on the assumption that SAGE tag counts follow a Poisson distribution, the PoissonC algorithm [1] models SAGE data by Poisson statistics and implements Poisson-based distances into the *k*-means clustering procedure, as summarized in Table S1.

Table S1 Poisson model-based *k*-means algorithm

1: **Initialise**: Determine the number of clusters, *k*.
Randomly assign all SAGE tags to *k* clusters.
2: **Repeat**
3: Find out the cluster profile for each cluster using joint Poisson distribution
4: Reassign each tag to the cluster using the chi-square statistics as a measure of the deviation
5: **Until** there is a convergence

S.2 Figure of Merits

In order to estimate the appropriate number of clusters, the *Figure of Merits* (FOM) was applied. Previous research has applied the FOM as an estimate of the predictive power of a clustering algorithm [2]. The method first relies on the application of a clustering algorithm to the data that represent all but one of the experimental conditions (a library in our case). Thus, based on the assumption that meaningful clusters of conditions (libraries) should exhibit less variation than clusters formed by chance, the condition left out is then used to assess the predictive power of the resulting clusters as explained elsewhere [2]. The lower the FOM value is, the higher the predictive power of the algorithm.

S.3 Clustering of SAGE tags with Euclidean distance

Fig. S1 shows the *k*-means clustering results with Euclidean distance on the normalised SAGE data. The results were generated using microarray the software suite, TM4, provided by the *Institute for Genomic Research* (TIGR) (http://www.tm4.org/mev.html) [4].

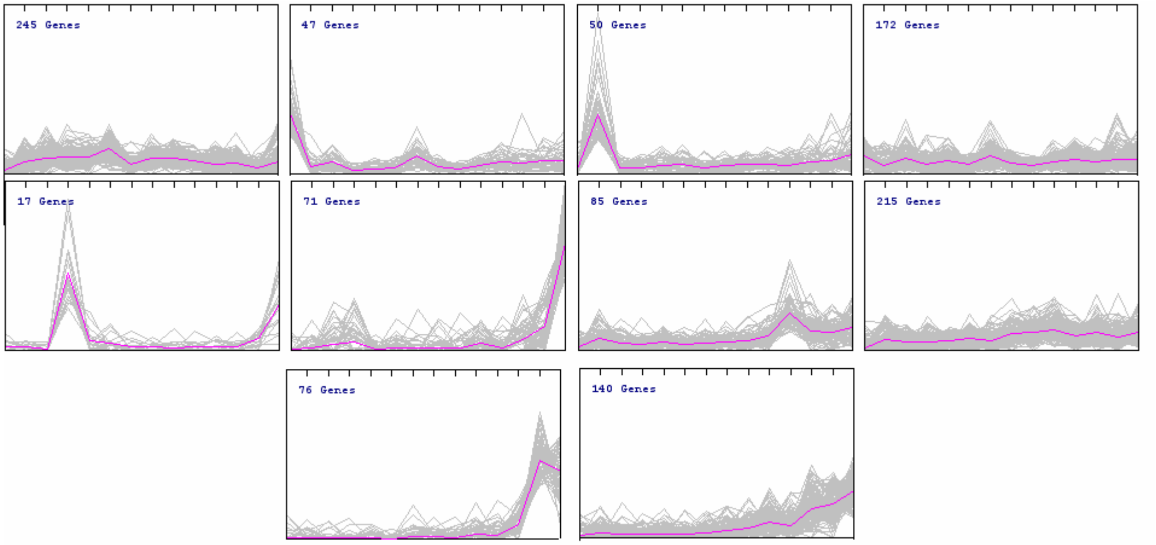

Fig. S1 Euclidean distance based *k*-means clustering analysis on the normalised SAGE data using 10 clusters. SAGE libraries are plotted on the x-axis. Numbers one to fourteen represent the fourteen SAGE libraries (1: hypo; 2: 3t3; 3: E12.5; 4: 14.5; 5: E16.5; 6:E18.5; 7: P0.5; 8: P2.5; 9: P4.5; 10: P6.5; 11: P10.5Crx-/-; 12:P10.5Crx+/+; 13: Adult; 14: ONL). Tag abundance is shown on the y-axis. Data were normalized before plotting. Each tag from the 14 libraries was rescaled to make the sum of the expression values equal to one. The pink line represents the median plot of each cluster.

S4. Projection-based data analysis

261 PR-enriched and 63 non-PR-enriched tags were projected onto 3-dimensional maps using well-known mapping methods: *Principal Component Analysis* (PCA) and *Sammon's mapping* [3], as illustrated in Fig. S2. These figures indicate that the two classes are not linearly separable from each other.

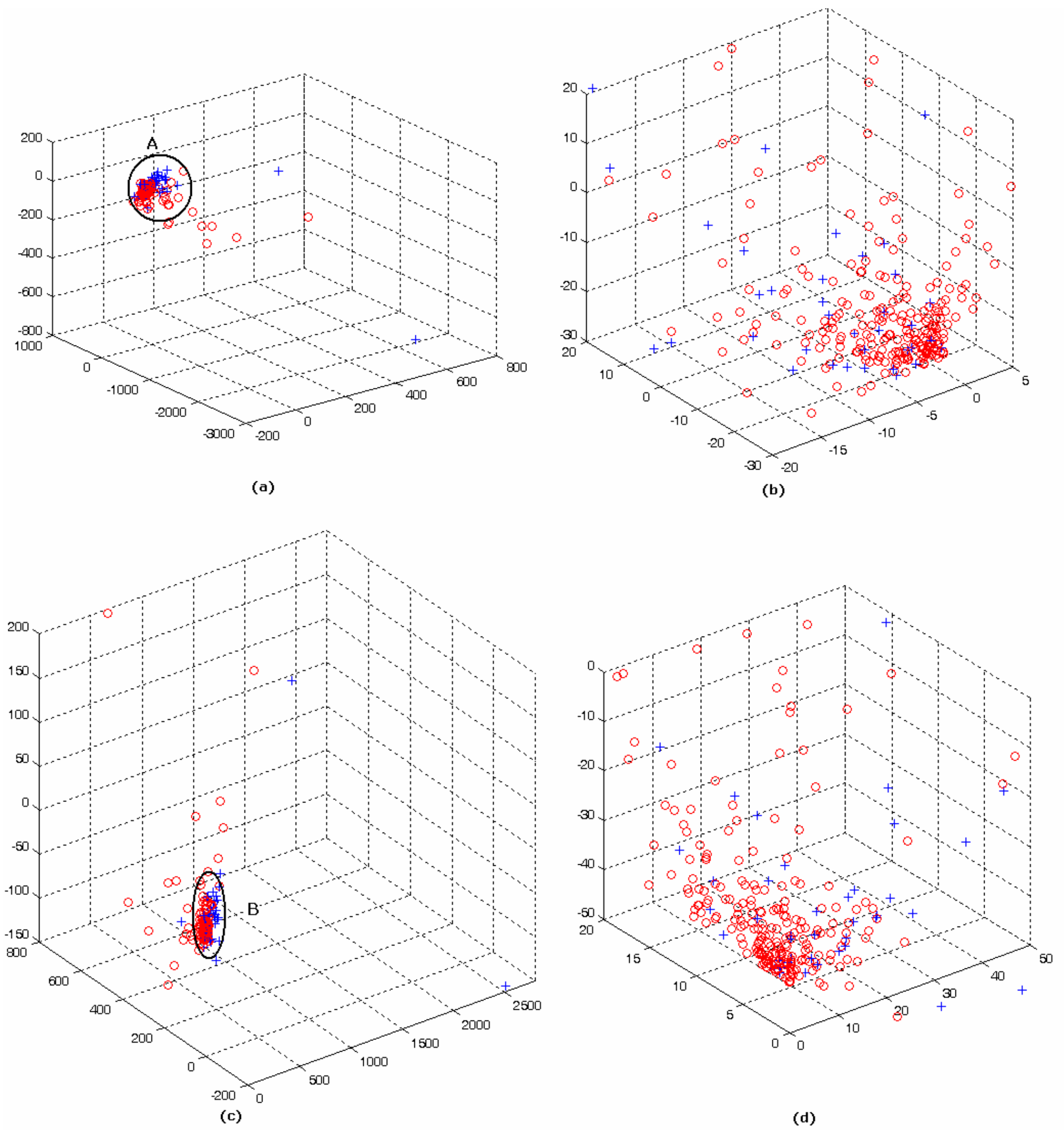

Fig. S2 Projection-based data analysis: the 324 tags are projected onto a 3D subspace with (a) Sammon's mapping; (c) Principal Component Analysis (PCA). Red "o" represents PR-enriched tags, and blue "+" stands for non-PR-enriched tags. (b) and (d) are zoomed images of marked areas A and B in (a) and (c) respectively. These plots indicate that the two classes are not linearly separable from each other.

S5. Criterion-based data analysis

The distribution of the 324 tags on the basis of their compliance with the four criterion introduced by Blackshaw *et al*. [5] is shown in Table S2.

Table S2 The distribution of 324 tags over four criteria proposed by Blackshaw *et al.* (2001)

Criterion	Number of Tags			
1	Yes	218	PR-enriched	196
			Non PR enriched	22
	No	106	PR-enriched	65
			Non PR enriched	41
2	Yes	175	PR-enriched	164
			Non PR enriched	11
	No	149	PR-enriched	97
			Non PR enriched	52
3	Yes	177	PR-enriched	149
			Non PR enriched	28
	No	147	PR-enriched	112
			Non PR enriched	35
4	Yes	234	PR-enriched	194
			Non PR enriched	40
	No	90	PR-enriched	67
			Non PR enriched	23

S6. Supervised classification methods

Three different classification models were implemented using the free available Weka package [6], [7]: Kstar, C4.5 tree, and *multilayer perceptron* (MLP) neural network model. KStar is an instance-based classifier, i.e. the class predicted for a test instance is based upon the class of those training instances similar to the test instance, as determined by some similarity function. It differs from other instance-based learners in that it uses an entropy-based distance function.

Unless indicated otherwise, the parameters used are given as follows. *Globalblend* for Kstar is set to 5. For C4.5 algorithm, the *confidence factor* used for pruning is 0.25 and the *minimum number of instances per leaf* is set to 2. The *learning rate* for MLP is 0.3, and the *momentum* applied to the weights during updating is equal to 0.2.

S7. Evaluation methods for supervised classification

Four metrics: precision (*Pr*), true negative rate (also known as *specificity, Sp*), true positive rate (also known as *sensitivity, Se*), and *Receiver Operating Characteristic* (ROC) graphs, were used in this study to assess the quality of a classifier. A ROC graph characterises the performance of classifiers across all possible predictive trade-offs between the false negative and false positive rates. The plot shows the false positive rate (1 – specificity) on the X axis and the true positive rate (sensitivity) on the Y axis. The closer the curve follows the left-hand border and then the top border of the ROC space,

the better the classifier's performance is. A ROC curve following the function $y = x$ indicates that a classifier's prediction performance is equivalent to making predictions based on random class assignments.

In the case of a two-class classification problem ($c = 2$), the *Se* and *Sp* are normally constructed based on the concept of *true positives, TP* (samples correctly classified as class *i*), *false negatives, FN* (samples incorrectly classified as not belonging to class *i*), *false positives, FP* (samples incorrectly classified as class *i*), *and true negatives, TN* (samples correctly classified as not belonging to class *i*), as illustrated in Table S3 and equations (1) to (3).

Table S3 An illustration of A confusion matrix

		Predicted	
		Positive	Negative
Actual	Positive	*TP*	*FN*
	Negative	*FP*	*TN*

$$Pr(\%) = \frac{TP}{TP + FP} \times 100\% \quad (1)$$

$$Se(\%) = \frac{TP}{TP + FN} \times 100\% \quad (2)$$

$$Sp(\%) = \frac{TN}{FP + TN} \times 100\% \quad (3)$$

S 8. Permutation test

100-run permutation tests were performed in this study, i.e.: the labels for each tag were randomly shuffled, classifiers were then implemented, their prediction quality was assessed and this process was repeated for a number of permuted datasets. By counting the times the permuted datasets produced better results than the classifier built on the original dataset, the statistical significance was then established. The complete results of the permutation test can be found in the additional file 3.

REFERENCES

1. Cai L, Huang H, Blackshaw S, Liu JS, Cepko C, Wong W: **Clustering analysis of SAGE data: A Poisson Approach**. *Genome Biology* 2004, 5: R51.
2. Yeung KY, Haynor DR, Ruzzo WL: **Validating clustering for gene expression data**. *Bioinformatics*, 2001, 17(4), 309-318.
3. Sammon JW: **A nonlinear mapping for data analysis**. *IEEE Transactions on Computers* , 1969, C-18, 401-409.

4. Saeed AI, Sharov V, White J, Li J, Liang W, Bhagabati N, Braisted J, Klapa M, Currier T, Thiagarajan M, Sturn A, Snuffin M, Rezantsev A, Popov D, Ryltsov A, Kostukovich E, Borisovsky I, Liu Z, Vinsavich A, Trush V, Quackenbush J: **TM4: a free, open-source system for microarray data management and analysis**. *Biotechniques*, 2004, 34(2), 374-378
5. Blackshaw S, Fraioli RE, Furukawa T, Cepko CL: **Comprehensive analysis of photoreceptor gene expression and the identification of candidate retinal disease genes**. *Cell* 2001, 107, 579-589.
6. Frank E, Hall M, Trigg L, Holmes G, Witten, IH: **Data mining in bioinformatics using Weka**. *Bioinformatics* 2004, 20, 2479-2481.
7. Witten IH, Frank E: *Data Mining: Practleical machine learning tools and techniques*, 2nd Edition, Morgan Kaufmann, San Francisco, 2005.
